# Molecular detection of *Bartonella* and *Borrelia* in pet dogs in Metro Manila and Laguna, Philippines

**DOI:** 10.14202/vetworld.2023.1546-1551

**Published:** 2023-07-31

**Authors:** Chae Eun Lee, Jeong Hee Ikeda, Mikaella Andrea M. Manongdo, Dan Rica T. Romerosa, Kristina Andrea C. Sandalo-De Ramos, Tetsuya Tanaka, Remil L. Galay

**Affiliations:** 1 Department of Veterinary Paraclinical Sciences, College of Veterinary Medicine, University of the Philippines Los Baños, College, Laguna 4031, Philippines; 2 Laboratory of Infectious Diseases, Joint Faculty of Veterinary Medicine, Kagoshima University, Korimoto 1-21-24, Kagoshima 890-0065, Japan

**Keywords:** *Bartonella*, *Borrelia*, dogs, polymerase chain reaction, tick-borne pathogens

## Abstract

**Background and Aim:**

*Bartonella* and *Borrelia* are zoonotic vector-borne pathogens that can infect dogs and humans. Data on *Bartonella* and *Borrelia* in dogs in the Philippines are lacking. This study was conducted to validate previous reports and further investigate the occurrence of *Bartonella* and *Borrelia* spp. in cities of Metro Manila.

**Materials and Methods:**

A total of 182 canine blood samples were acquired with DNA using a commercial extraction kit from selected veterinary clinics in the cities of Metro Manila and Laguna, Philippines. The mammalian *actin* was amplified through polymerase chain reaction (PCR), followed by PCR assays targeting *Bartonella* *gltA* and *Borrelia* *flaB*. Further PCR assays targeting *16S* of *Borrelia* and *ospA* and *ospC* of *Borrelia burgdorferi* were performed for those that showed *flaB* bands.

**Results:**

A positive band for *gltA* of *Bartonella* was observed in 9 (4.95%) samples, whereas a positive band for *flaB* of *Borrelia* was observed in 15 (8.24%) samples. Subsequent PCR assays for other genes of *Borrelia* were negative.

**Conclusion:**

These results confirmed the presence of *Bartonella* and warranted further investigation for the possible presence of other *Borrelia* species.

## Introduction

Dog ownership is increasingly popular in the Philippines, which is among the top countries with a high dog population estimated at 23 million [1] and has the highest dog ownership rates in Asia [2]. Because many dog owners live in close contact with their pets, there is a risk of zoonotic transmission of some diseases. Tick infestation and tick-borne diseases are common among dogs in the Philippines [3]. Thus, zoonotic vector-borne diseases affecting dogs can significantly threaten public health.

*Bartonella* is a zoonotic vector-borne pathogen distributed worldwide and can infect various mammals, including humans. Several *Bartonella* species cause human diseases that can be acquired from mammalian reservoir hosts [4]. Only a few studies have reported clinical bartonellosis in dogs [5, 6]. Domestic dogs can be infected with several *Bartonella* species and are considered reservoirs of those that are pathogenic or potentially pathogenic to humans [7]. In a recent study in the Philippines, *Bartonella henselae* DNA was detected in apparently healthy dogs and those showing clinical signs [8]. In another study, *Ctenocephalides* flea samples from dogs in Metro Manila were positive for *Bartonella clarridgeiae* DNA [9]. Meanwhile, borreliosis is a zoonotic infection caused by gram-negative spirochetes of the genus *Borrelia*, divided into two taxonomic groups: The Lyme disease agents or *Borrelia burgdorferi* sensu lato (s.l.) complex and the tick-borne relapsing fever agents [10–12]. Information on *Borrelia* in Southeast Asia, particularly in the Philippines, is lacking. *Borrelia* is presently not a pathogen of concern in the Philippines, and borreliosis is excluded in differential diagnoses of most veterinary practitioners in the country. However, some veterinarians encountered cases of dogs being seropositive for *B. burgdorferi* s.l. based on a rapid antibody test kit. Furthermore, a recently published study reported a dog from the Philippines that was seropositive for *B. burgdorferi* s.l. using the same test kit [13]. Due to the limited data available on these zoonotic vector-borne pathogens of dogs in the Philippines, there is a need to conduct additional studies to confirm their presence and assess the potential risk to dog owners. Extensive knowledge of *Bartonella* and *Borrelia*, including its epidemiology in dogs, is crucial in the diagnosis of the diseases they cause, enabling the administration of treatment and implementation of necessary control measures.

This study aimed to confirm *Bartonella* and *Borrelia* in pet dogs in Metro Manila and Laguna, Philippines, by detecting pathogenic DNA using polymerase chain reaction (PCR). We aimed to provide additional information on the geographic distribution of these zoonotic vector-borne pathogens, particularly in South-east Asia and the Philippines, to raise awareness among veterinarians and dog owners, facilitating necessary prevention and control measures employing the One Health approach.

## Materials and Methods

### Ethical approval

The collection of blood samples was approved by the University of the Philippines Los Baños Institutional Animal Care and Use Committee with approval number UPLB-2021-036.

### Study period and location

The study was conducted from July 2021 to December 2022. The study covered the National Capital Region of the Philippines (Metro Manila) and the nearby province of Laguna due to the high population of dogs in these areas. Veterinary clinics/hospitals in selected cities or municipalities were recruited to collect blood from pet dogs.

### Collection of blood samples

Veterinarians in participating veterinary establishments were requested to collect at least 0.5 mL blood from dogs with existing or previous tick infestation within the past 12 months with or without clinical signs of tick-borne diseases at the time of presentation, regardless of sex, age, and breed. The dog owners were requested to sign a consent form to obtain permission for blood sample collection and the participation of their dogs in the study. A questionnaire on the signalment and medical history of the dogs was also completed. A total of 182 blood samples were retrieved and transported in an ice pack (4ºC) to the Veterinary Molecular Biology Laboratory at the Department of Veterinary Paraclinical Sciences, College of Veterinary Medicine, University of the Philippines Los Baños, Laguna, where the samples were stored at –20°C till further use.

### DNA extraction and detection of control genes

A commercial spin column-based extraction kit was used to extract DNA (GF-1 Total DNA Extraction Kit®, Vivantis, Selangor, Malaysia) according to the manufacturer’s protocol. To ensure successful DNA extraction, the DNA samples were subjected to a conventional PCR targeting mammalian *actin* using the Tks Glex® polymerase kit (Takara, Shiga, Japan) with the primers and conditions previously described by Galay *et al*. [3].

### Polymerase chain reaction assays for detection of *Bartonella* and *Borrelia*

After successful amplification of *actin*, all 182 canine blood DNA samples were subjected to PCR assays to detect *Bartonella* and *Borrelia*. Conventional PCR targeting *gltA* was performed to detect *Bartonella* using the primers BhCS.781p and BhCS.1137n [14], as described by Singer *et al*. [8]. Meanwhile, nested PCR targeting *flaB* of *Borrelia* was initially performed using the primers and conditions described by Loh *et al*. [15]. For samples that showed a positive band for *flaB*, PCR assays targeting *16S* of *Borrelia* [15] and *ospA* and *ospC* genes of *B. burgdorferi* [16] were further performed. A negative control containing sterile water as a template was included in each PCR run. Following PCR, gel electrophoresis was performed using 1.5% agarose gel, and the bands were visualized after staining the gel with ethidium bromide (1 μg/mL) in Tris-HCl, acetic acid, ethylenediaminetetraacetic acid buffer. The positivity rates were calculated by dividing the positive samples by the tested samples and expressed as a percentage.

## Results

**Figure-1:**
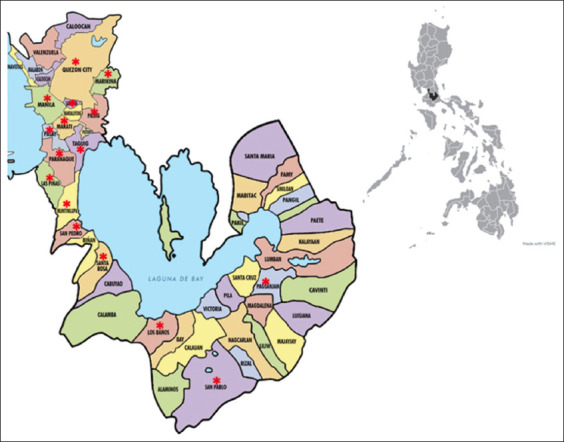
Map showing the study area. The smaller map shows the location of Metro Manila and Laguna (shaded black) in the Philippines. The larger map shows the localities (marked with asterisk) where the veterinary establishments are located. The small map was prepared using the online graphic design software Visme (https://www.visme.co/).

The 182 canine blood samples were retrieved from veterinary establishments in 12 cities of Metro Manila and 5 cities/municipalities in Laguna (Figure-1). The information on the attributes of the dogs gathered through the questionnaires is reviewed and summarized in Table-1. There were more blood samples from male dogs than female dogs, although there were six dogs whose data on sex were not indicated. Most samples came from dogs >3 years old. The majority of dogs were purebred dogs. During blood collection in veterinary clinics/hospitals, 50 (27.47%) dogs had existing tick infestation, whereas 97 (53.3%) had previous infestation within the past 12 months. Regarding the health status, most dogs (48.35%) showed non-specific clinical signs (e.g., fever, vomiting, anorexia, weakness, and lethargy) or clinical manifestations related to tick-borne infection (e.g., bleeding tendencies, anemia, and thrombocytopenia). At the attending veterinarian’s discretion, some patients were also tested for antibodies against common tick-borne pathogens using commercial rapid antibody detection kits as part of their diagnostic procedure. Several samples tested positive for antibodies against *Ehrlichia canis* and/or *Anaplasma platys*. Moreover, two samples tested positive for *B. burgdorferi* s.l. antibody. Most dogs (n = 80) showing clinical manifestations were treated with doxycycline.

**Table-1:** Select attributes of the 182 dogs from Metro Manila and Laguna, Philippines that were tested for *Bartonella* and *Borrelia* using PCR. The distribution of samples positive for *Bartonella gltA* and *Borrelia flaB* genes according to attributes is also shown.

Attribute	Number of dogs	Number (%) positive for *Bartonella gltA*	Number (%) positive for *Borrelia flaB* gene
Sex			
Male	97	3 (3)	8 (8.2)
Female	79	6 (7.6)	7 (8.9)
No data	6	0	0
Age			
Puppy (<1 year)	22	1 (4.5)	0
Juvenile (1–3 years)	45	2 (4.4)	3 (6.7)
Adult (>3 years)	103	(4.9)	12 (11.7)
No data	12	1 (8.3)	0
Breed			
Pure	105	7 (6.7)	10 (9.5)
Mixed	67	1 (1.5)	5 (7.5)
No data	10	1 (10)	0
Tick infestation			
Existing	50	6 (12)	7 (14)
Previous (within 12 months)	98	2 (2)	6 (6.1)
No data	35	1 (2.9)	2 (5.7)
Health status			
With clinical signs	89	5 (5.6)	11 (12.4)
Apparently healthy	12	3 (25)	4 (33.3)
No data	81	1 (1.2)	0

**Figure-2:**
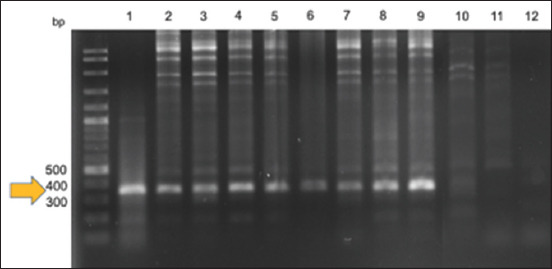
Representative agarose gel photograph after polymerase chain reaction for *Bartonella* spp. targeting the *gltA* gene in representative blood samples from dogs. Lanes 1–9 are positive samples showing the band of around 380 bp, as pointed by an arrow; lanes 10 and 11 are negative samples; lane 12 is the negative control (sterile nuclease-free water as a template).

After DNA extraction, the control actin gene was successfully amplified from all 182 samples. For *Bartonella* detection, 9 (4.95%) samples showed a positive band for *gltA* with a size of ~380 bp (Figure-2). The positive samples came from dogs belonging to six Metro Manila and Laguna localities. Three positive samples came from male dogs, whereas six positive samples were from female dogs (Table-1). Most *Bartonella*-positive samples came from pure-breed dogs (7/9) and those <1 year old (8/9). Most *Bartonella*-positive dogs were currently (2/9) or previously (6/9) infested with ticks. Moreover, most of these *Bartonella*-positive dogs (6/9) exhibited clinical signs, such as fever, inappetence, lethargy, pale mucous membrane, weakness, weight loss, and vomiting during presentation. Hematological abnormalities, including leukocytosis (5/9), thrombocytopenia (3/9), or anemia (two of nine), were also reported. Two *Bartonella*-positive dogs did not show any clinical signs, and one lacked information on the health status.

In contrast, 15 samples had a band of ~407 bp after nested PCR for *flaB* of *Borrelia*. Most samples came from dogs that showed non-specific clinical signs and had existing or previous infestations with ticks. One of the *Borrelia* *flaB*-positive samples tested faintly positive for *B. burgdorferi* s.l. antibody based on a rapid test kit. The 15 samples were subjected to PCR assays targeting *16S* of *Borrelia* and *ospA* and *ospC* of *B. burgdorferi*. However, none showed a positive band in any of these target genes. No codetection of *Bartonella gltA* and *Borrelia flaB* was observed.

## Discussion

The occurrence of tick-borne pathogens that primarily cause diseases in dogs, such as *A. platys*, *E. canis*, *Babesia vogeli*, and *Hepatozoon canis*, has been well documented in the Philippines through studies using molecular techniques [3, 17–19]. However, data on zoonotic vector-borne pathogens *Bartonella* and *Borrelia* are lacking despite anecdotal reports of some veterinarians on detecting antibodies against the latter based on a rapid test kit.

This study provided additional molecular evidence of the occurrence of *Bartonella* in dogs in the Philippines, supporting the recent report of Singer *et al*. [8] that covered only two veterinary clinics in Metro Manila. Similar to the previous report, most dogs found positive for *Bartonella* DNA exhibited clinical signs. Thrombocytopenia was observed in three *Bartonella* DNA-positive dogs, a hematological finding in dogs infected with *Bartonella* based on previous reports by Singer *et al*. [8], Breitschwerdt *et al*. [20], Baneth *et al*. [21]. Anemia was observed in two dogs, similar to reports on seropositive dogs for *Bartonella vinsonii* subsp. *berkhoffii* [20]. As reported by the attending veterinarians, three of these *Bartonella* DNA-positive dogs were seropositive for *E. canis*. Thus, it was uncertain whether *Bartonella* directly caused the clinical disease or aggravated *E. canis* infection. Moreover, concurrent infection with tick-borne pathogens among dogs has been previously confirmed in the Philippines by Galay *et al*. [3]. Thus, it is highly possible that *Bartonella* could be involved in concurrent infections and may aggravate the dog’s clinical condition, as previously reported by Breitschwerdt *et al*. [5].

Nested PCR for *flaB* was performed as a screener because it is highly conserved among various genotypes of *Borrelia*. This screening assay targeting a major flagellum filament gene is efficient due to its high sensitivity and the presence of *flaB* in the Lyme disease complex and the relapsing fever group [22]. However, in this study, the samples positive for *flaB* did not show positive bands in subsequent PCR assays for *Borrelia* and *B. burgdorferi* s.l. The negative PCR results for *ospA* and *ospC* suggested that the samples were not infected with Lyme disease agents, including the one seropositive sample, based on a commercial antibody test kit. Moreover, none of the dogs showing a positive band for *Borrelia flaB* exhibited any specific clinical signs attributable to borreliosis. A study in Thailand reported a very low detection rate of *B. burgdorferi* s.l., wherein only one of 402 dogs tested positive in PCR assays targeting the three genes that were also analyzed in this study [16]. In that report of Thailand, one PCR-positive dog was seropositive for *B. burgdorferi* s.l. based on a commercial test kit, which contrasts with the results of this study. In contrast, ongoing treatment with doxycycline in some dogs might interfere with the detection of *Borrelia* and *Bartonella* as this antibiotic can reduce the bacterial load in the animal’s blood.

Most dogs that tested positive for *Bartonella* DNA in this study had ticks at the time of clinical presentation or had previous tick infestation within the past 12 months. The brown dog tick *Rhipicephalus sanguineus* s.l. is widely distributed in the Philippines and primarily responsible for tick-borne diseases spread in dogs [3]. A recent study demonstrated the potential role of *R. sanguineus* s.l. ticks in the transmission of *B. henselae* [23]. Thus, dogs in this study might have acquired *Bartonella* from ticks. Moreover, infestation with *Ctenocephalides* fleas is also quite common among dogs in the Philippines, and a recent study reported the detection of *Bartonella* DNA from fleas collected through veterinary clinics in Metro Manila [9]. Regarding the vector of *Borrelia*, the presence of proven vector ticks under the genus *Ixodes* in the Philippines has not been confirmed. *Ixodes granulatus* is a potential vector of *Borrelia* reported in Southeast Asian countries, such as Malaysia, Singapore, Taiwan, and Laos [24–26]. However, there has been no recent documentation or anecdotal reports of *I. granulatus* in dogs in the Philippines. The potential role of *R. sanguineus* in the transmission of *Borrelia* has not yet been proven despite reports on molecular detection of the pathogen’s DNA in the tick [27, 28]. As the presence of *Ixodes* in dogs is not reported, there is a low possibility for the active transmission of *B. burgdorferi* s.l. in the study area and a low risk for zoonotic transmission.

## Conclusion

This study detected *Bartonella* in dogs in Metro Manila and Laguna using PCR. Although some blood samples tested positive for *flaB* of *Borrelia*, the negative results of subsequent PCR assays, which targeted other genes, cannot confirm the presence of the pathogen. This study provided additional epidemiological data on *Bartonella* in dogs in the Philippines and South-east Asia. Further studies in the other areas of the Philippines with a large sample size including stray dogs are recommended to estimate the prevalence and geographical distribution of *Bartonella*. Despite the negative results for *Borrelia*, further surveillance is necessary for safety against its possible emergence in the country. Further studies on other *Borrelia* species and the detection in arthropod vectors are also recommended to facilitate targeted control of its transmission. Increasing the awareness of veterinarians, dog owners, and medical practitioners on these zoonotic vector-borne pathogens is a crucial aspect of the one health approach in controlling these diseases.

## Authors’ Contributions

CEL, JHI, MAMM, DRTR, and KACS: Performed the sample collection, experiments, and data analysis. CEL, JHI, MAMM, and DRTR: Prepared the initial draft of the manuscript. RLG and TT: Conceptualized the study and acquired funding. RLG: Supervised the implementation of the study, verified data analysis, and finalized the manuscript. All authors have read, reviewed, and approved the final manuscript.
